# Supplementary material for: Association between oral microbiome and seven types of cancers in East Asian population: a two-sample Mendelian randomization analysis
Source: Front Mol Biosci. 2023 Nov 21;10:1327893. doi: 10.3389/fmolb.2023.1327893 (PMC10702768; doi:10.3389/fmolb.2023.1327893)
Supplement: Supplementary file 1 [file DataSheet1.ZIP › Supplementary Materials/MR plots of saliva/saliva═╝/Pancreatic cancer/pheno.1031_to_pancreatic cancer_leave_one_out.pdf]

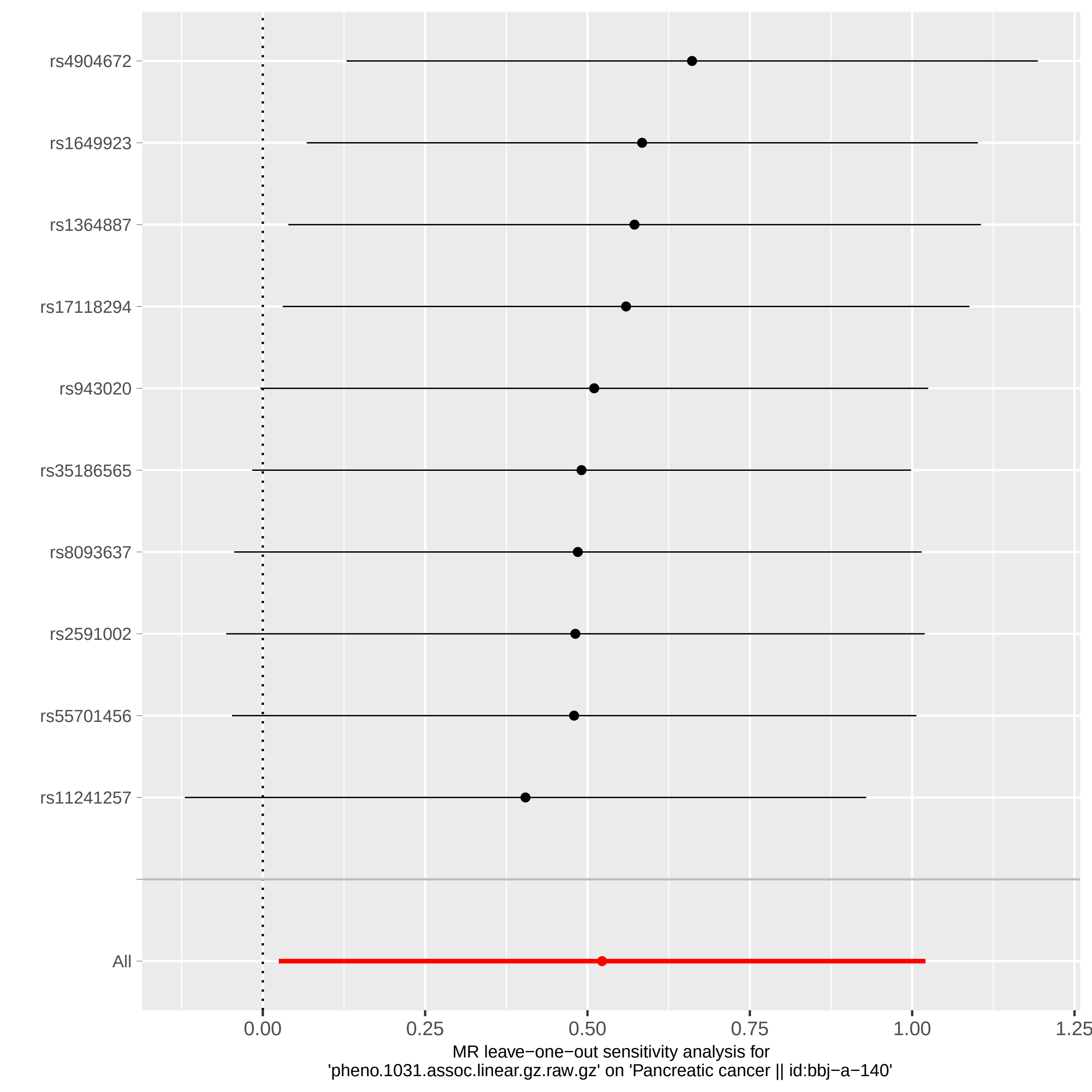
rs4904672
rs1649923
rs1364887
rs17118294
rs943020
rs35186565
rs8093637
rs2591002
rs55701456
rs11241257
All
0.00
0.25
0.50
0.75
1.00
1.25
MR leave−one−out sensitivity analysis for
'pheno.1031.assoc.linear.gz.raw.gz' on 'Pancreatic cancer || id:bbj−a−140'
